# Near-Infrared Spectroscopy for the Evaluation of Anesthetic Depth

**DOI:** 10.1155/2015/939418

**Published:** 2015-10-01

**Authors:** Gabriela Hernandez-Meza, Meltem Izzetoglu, Mary Osbakken, Michael Green, Kurtulus Izzetoglu

**Affiliations:** ^1^School of Biomedical Engineering, Science and Health Systems, Drexel University, 3508 Market Street, Suite 100, Philadelphia, PA 19104, USA; ^2^Department of Anesthesiology, Drexel University College of Medicine, Hahnemann University Hospital, 245 N. 15th Street, MS 310, Philadelphia, PA 19102, USA

## Abstract

The standard-of-care guidelines published by the American Society of Anesthesiologists (ASA) recommend monitoring of pulse oximetry, blood pressure, heart rate, and end tidal CO_2_ during the use of anesthesia and sedation. This information can help to identify adverse events that may occur during procedures. However, these parameters are not specific to the effects of anesthetics or sedatives, and therefore they offer little, to no, real time information regarding the effects of those agents and do not give the clinician the lead-time necessary to prevent patient “awareness.” Since no “gold-standard” method is available to continuously, reliably, and effectively monitor the effects of sedatives and anesthetics, such a method is greatly needed. Investigation of the use of functional near-infrared spectroscopy (fNIRS) as a method for anesthesia or sedation monitoring and for the assessment of the effects of various anesthetic drugs on cerebral oxygenation has started to be conducted. The objective of this paper is to provide a thorough review of the currently available published scientific studies regarding the use of fNIRS in the fields of anesthesia and sedation monitoring, comment on their findings, and discuss the future work required for the translation of this technology to the clinical setting.

## 1. Introduction

In the United States, awareness with recall during general anesthesia has been estimated to occur in 1-2 cases per 1000 patients annually, which amounts to approximately 26,000 cases per year [1–3]. The classifications of events that constitute awareness are somewhat controversial and can range from feelings of pain to recall of conversations during surgery. In other countries, such as China, the incidence of anesthesia awareness has been found to be as much as two times higher than that of the United States [4], while a British audit found the incidence to be lower [5]. The studies conducted thus far have linked awareness with recall to age, female sex, obesity, anesthetist's experience, length of surgery, and previous awareness events. Patients who have suffered from intraoperative awareness have been known to experience long term psychological consequences, most notably posttraumatic stress disorder [6].

Anesthetic agents used during general anesthesia disrupt the activity of neurons in a dose dependent manner in order to suppress memory formation and awareness. This decrease in neural activity can be visualized with positron emission tomography (PET) as a decrease of the glucose metabolic rate (GMR) and the cerebral metabolic rate of oxygen (CMRO_2_) consumption [7–9]. Furthermore, it is to be noted that all volatile anesthetics suppress the cerebral metabolic rate and affect the cerebral blood flow in a dose dependent manner [10]. This information is very important, due to the fact that the cortical regions of the brain, including the prefrontal cortex, experience functional inhibition during anesthesia [11–14].

Under the current standard of care published by the ASA, continuous monitoring of oxygenation, ventilation, circulation, and temperature is required [15]. These measurements allow the anesthesia provider to detect adverse events and improve patient safety during surgery; however, they are not specific to the effect of the anesthetics on the brain. The lack of a “gold-standard” method for assessing the effects of anesthetics and sedatives on the brain has led to the investigation of electroencephalographic (EEG) signals, auditory evoked potential (AEP), and facial electromyography (EMG) signals as possible monitors of depth of anesthesia. Currently, devices based on these technologies, such as the Bispectral Index (BIS) monitor (Medtronic, Dublin, Ireland), are clinically available. The BIS monitor evaluates the Fourier domain of electroencephalograph epochs and through a proprietary algorithm outputs a number between 0 and 100, with values under 60 representing the anesthetized state. Still, although accessible, the routine use of EEG monitors has been estimated to be approximately 1.8% [16]. The low rate of use of these devices may be influenced by reduced performance of the devices in the presence of opioids [17] and the fact that they have been found to provide information that correlates with the patient's state but are not predictive enough to guide the delivery of anesthetics [18, 19]. In contrast to other means of monitoring depth of anesthesia, near-infrared spectroscopy (NIRS) monitors change in circulatory oxygenation of the cerebral cortex, which can reflect tissue oxygen use [20, 21]. These facts guided the reasoning for investigating the sensitivity of hemodynamic response measured by functional near-infrared spectroscopy (fNIRS) on the prefrontal cortex (PFC) to determine changes in the anesthetic depth.

fNIRS is a safe, noninvasive, and portable optical method that can be used to monitor activity within the cortical areas of the human brain. Making use of specific wavelengths of light, fNIRS provides measurements of oxygenated (HbO_2_) and deoxygenated (Hb) hemoglobin that are in direct relation with hemodynamic changes in the brain [20–22]. A close link between hemodynamics and neural activity has been established through brain-energy metabolism research [23–25]. Previous studies comparing fNIRS to established neuroimaging technologies, such as functional magnetic resonance imaging (fMRI) and PET, have shown that fNIRS provides comparable results in various domains including attention, memory, and sensory/motor areas in healthy and diseased/disabled populations of various age groups [26–30].

fNIRS has also been used to detect cerebral hypoxia during carotid endarterectomy [31, 32] and to monitor cerebral perfusion during liver transplantation [33]. Its capacity to determine the hemodynamic changes that occur in the cerebral cortex as a consequence of anesthetic use makes this technology an ideal candidate for the study and development of monitors for anesthetic depth. Furthermore, because it is portable and noninvasive, fNIRS can be of substantial practical use in clinical settings.

The primary purpose of this paper is to provide a review of studies of anesthesia monitoring using near-infrared spectroscopy to provide a better understanding of the relationship between measurable hemodynamic changes in the prefrontal cortex and changes in anesthetic state. This review also presents information on the challenges associated with the translation of this technology into a clinical environment. This review will begin by outlining the results of investigations of anesthetic drug effects on cerebral metabolism and hemodynamics, continue with studies that evidence potential confounding factors for the fNIRS signal, and conclude with studies that have uncovered potential biomarkers in the fNIRS signal that could signal changes in anesthetic depth.

## 2. Methods

The study selection was performed using the PubMed database to find studies that investigated brain hemodynamics during anesthesia or sedation using functional near-infrared spectroscopy. The search was restricted to English language articles published between January 1, 1990, and May 1, 2015. The search terms included “functional near infrared spectroscopy,” “fNIR,” “NIRS,” “anesthesia,” “sedation,” and “neuromarker.” During the search, journal articles with topics related to oxygen saturation monitoring during cardiac or other surgeries were excluded. The results of the studies were divided into three groups: (i) findings on the effects of anesthetics on cerebral hemodynamics, (ii) findings related to confounding factors of the fNIRS signal, and (iii) findings on fNIRS biomarkers of depth of anesthesia. Tables 1–3 contain a summary of the findings of the studies presented in this paper.

To further inform the reader on the topics examined in this review we present representative example plots from our data obtained during a study of 50 patients undergoing elective surgery at the Hahnemann University Hospital, Philadelphia, PA. The study protocol and statements of informed consent were approved by the Institutional Review Board (IRB) of Drexel University. The study was conducted with the subjects' understanding and consent. The figures presented in this review originated from fNIRS data recorded during three elective surgical procedures, of patients classified as ASA class I or II, under general anesthesia using propofol for induction and sevoflurane for maintenance. In this review, data from this study was used to serve as examples, when relevant, for the results described in previous studies.

## 3. Results

The results of the literature search were grouped by the following topics: (i) findings on the effects of anesthetics on cerebral hemodynamics, (ii) findings related to confounding factors of the fNIRS signal, and (iii) findings on fNIRS biomarkers of depth of anesthesia.

### 3.1. Effects of Anesthetics on Cerebral Hemodynamics

Initial studies of fNIRS in the area of anesthesia examined primarily the differences in cerebral metabolism and hemodynamics elicited by different drugs on the prefrontal cortex. These investigations focused on examining cerebral oxygen saturation (rSO_2_), cerebral blood volume as approximated by the total hemoglobin concentration (HbTot), and cerebral blood flow (CBF) in response to the administration of anesthetics. For clarity, this review separates the studies into two categories: (i) evaluation of fNIRS data during induction and (ii) evaluation of fNIRS data during steady state anesthesia maintenance. A summary of the findings of studies included in this section can be found in Table 1.

#### 3.1.1. Effect of Anesthesia Induction

Anesthetic drugs are known to decrease the CMRO_2_ and to have a drug and dose dependent effect on CBF. The examination of fNIRS derived hemodynamic changes during the induction of anesthesia provides a method for examining the effects of different drugs during the transition between the awake and anesthetized state.

**Figure 1:**
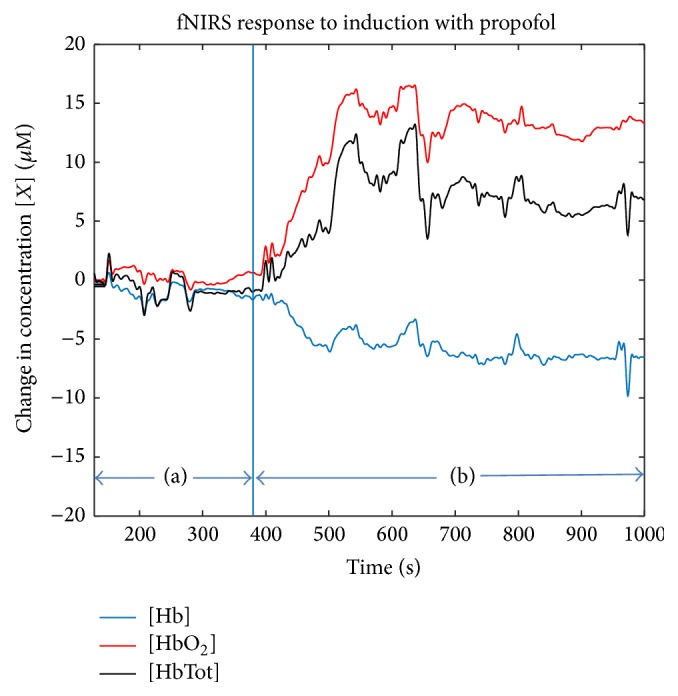
Effect of bolus propofol induction (200 mg) on [Hb], [HbO_2_], and [HbTot]. (a) Time before induction and (b) time after induction. Data collected from a 31-year-old female patient undergoing a laparoscopic hysterectomy. (G. Hernandez-Meza, personal communications, July 22, 2015, School of Biomedical Engineering, Science and Health Systems at Drexel University, Philadelphia, PA 19104).

Using fNIRS, Lovell et al. documented small changes in cortical brain oxygenation during the induction of anesthesia with propofol, thiopental, or etomidate by following the adult patient's hemodynamic changes from a baseline period until 3 minutes after the administration of the intravenous (IV) drugs [34]. Changes in oxygenation were reported as differences in the relative HbO_2_ and HbTot concentrations. This study found that propofol and thiopental induce an increase in both HbO_2_ and HbTot relative to the baseline period [34]. In our 50-patient study, we have observed similar changes in HbO_2_ and HbTot following propofol induction.  Figure 1 shows an example of the changes in oxygenation that occur after induction of anesthesia with propofol. In contrast, etomidate exhibited a decrease in both HbO_2_ and HbTot parameters, possibly due to the different effect of this drug on oxygen saturation and blood flow [34]. This study revealed the capacity of fNIRS to measure drug dependent changes in oxygenation associated with anesthetic induction. A similar study on a pediatric population examined the effects of propofol induction on cerebral oxygenation of the frontal cortex [35]. As in the study by Fleck et al., propofol was found to produce an increase in HbO_2_ and HbTot relative to the conscious period [35].

Iwasaki et al. examined the changes in HbO_2_ and HbTot that occur after induction with 67% N_2_O and propofol, sevoflurane at 5%, or sevoflurane at 8% [36]. The patient's hemodynamic changes were continuously recorded from preinduction period until three minutes after intubation. This study reported significant increases in HbO_2_ and HbTot after 8% sevoflurane induction compared to 5% sevoflurane and propofol [36]. This suggests that fNIRS is sensitive to changes caused by different concentrations of the same anesthetic. This is evidenced by the difference seen between 5% and 8% sevoflurane. However, this study does not match the findings of the other propofol induction studies outlined above. The results could be explained by the use of N_2_O in conjunction with propofol during the induction procedure, which differs from the method employed in the studies by Lovell et al. and Fleck et al.

These initial studies provided the ground work to demonstrate that fNIRS is capable of monitoring real time changes that occur as a consequence of the administration of anesthetics. Additionally, it was shown that this technology is capable of distinguishing between the effects of induction with different drugs, such as propofol, etomidate, and thiopental [34]. Different anesthetic concentrations of the same drug, such as 5% versus 8% sevoflurane, also registered different fNIRS signal signatures during induction [36].

#### 3.1.2. Evaluations during Anesthesia Maintenance

Initial studies of fNIRS parameters during maintenance focused on examining differences in rSO_2_ and CBF between anesthetics to understand the effect of anesthetics on the oxygen supply demand balance and metabolism. Differences in oxygen saturation on the frontal cortex were found between sevoflurane and propofol during maintenance of anesthesia [37]. Sevoflurane was found to produce higher rSO_2_ values, which indicated that sevoflurane at 1.5% maintains the CBF/CMRO_2_ coupling to a similar extent as propofol [37]. Measurements of rSO_2_ have also been used to evaluate the incidence of cerebral desaturation events during surgical procedures [37, 41]. An investigation of such events in adults between the ages of 18 and 65 during propofol and sevoflurane anesthesia found that desaturation occurred only during the use of propofol [37]. On the other hand, during a sevoflurane study of elderly patients cerebral desaturation occurred at 26% of the examined cases [41].

A study by Fassoulaki et al. [38] examined differences on rSO_2_ between sevoflurane and desflurane during maintenance. First, rSO_2_ was examined during maintenance with BIS values between 40 and 50. No difference in oxygen saturation was found between the inhalants desflurane and sevoflurane [38]. Next, rSO_2_ was measured during maintenance of BIS values between 20 and 30 by increasing the concentrations of the inhalants. Again, no significant difference in rSO_2_ between inhalants was found [38]. However, this study found that rSO_2_ was higher when BIS values were maintained between 20 and 30 than the ones obtained when BIS values were kept between 40 and 50 [38]. During the 15 minutes of the measurement at steady state maintenance, the rSO_2_ level was stable [38]. The study by Fassoulaki et al. [38] provided evidence that rSO_2_ changes with increasing concentrations of sevoflurane and desflurane.

Kanemaru et al. [39] examined the effect of administration of midazolam, isoflurane, or aminophylline during maintenance of anesthesia with propofol. The goal of this study was to determine if the BIS index changes that occur with additional drug infusion are mirrored by changes in oxygenation. It was found that the three previously mentioned drugs affected the BIS index but not rSO_2_, suggesting that they may only have a negligible effect on oxygenation [39].

fNIRS has also been employed to examine cerebral blood flow during anesthesia maintenance. A study quantifying CBF during steady state anesthesia maintenance with isoflurane versus conscious patients showed no difference between the two states [40].

The previously outlined studies offer evidence for the capacity of fNIRS to measure differences in oxygenation between types and concentrations of anesthetics that could help in the assessment of anesthetic depth [37, 38]. Moreover, the studies demonstrated that some anesthetic drugs (midazolam, aminophylline, and isoflurane) may affect anesthetic depth, while having no effect on oxygen saturation [39]. To determine the effects of midazolam, aminophylline, and isoflurane on brain oxygenation an investigation of all the fNIRS derived measures (i.e., Hb, HbO_2_, and HbTot) is necessary. Additionally, the studies indicate that oxygenation remains constant when the depth of anesthesia is maintained at a steady state [38].

### 3.2. Findings on Confounding Factors of the fNIRS Signal

Several factors can affect the oxygen supply demand balance during fNIRS measurements of patients under anesthesia, affecting the interpretation of the hemodynamic variables recorded by the device. It is important to understand how the fNIRS signal can be affected by these confounding factors so that algorithms can be developed to suppress the effect of confounding information. These factors can be related to the position, type of surgery, hematocrit levels, and ventilation. A summary of the findings of studies included in this section can be found in Table 2.

#### 3.2.1. Effects of Position

Position change, such as various tilt angles of the operating table, has been demonstrated to affect the hemodynamic parameters measured by fNIRS [42–46]. The hemodynamic changes observed after position change may be related to changes in CBV associated with the effect of gravity but are also dependent on the anesthetic. Changing to the Trendelenburg position after CO_2_ pneumoperitoneum was evaluated during propofol and sevoflurane anesthesia. Significant increase in rSO_2_ after CO_2_ insufflation and position change was present with the use of sevoflurane but not propofol [42]. This study also found lower rSO_2_ values for propofol anesthesia [42].

Changing to the sitting position was evaluated during anesthesia maintenance with propofol and desflurane anesthesia [43]. Upon shifting to the sitting position, both groups experienced a significant decrease in rSO_2_. The propofol group had significantly lower rSO_2_ values [43]. Oxygen saturation was measured during nine minutes in the sitting position and during this time no significant differences were observed within the groups. Returning to supine position restored rSO_2_ values to the baseline level [43]. The results show that moving to the sitting position caused a decrease in cerebral oxygen saturation in equipotent concentrations of propofol and desflurane groups, with propofol experiencing a larger decrease [43]. Similarly, movement from supine to beach chair position (BCP) was associated with a 10% decrease in rSO_2_ that was reversible after returning to the supine position [44]. Head-up position was also evaluated by Kitajima et al. [45] during maintenance with sevoflurane combined with 66% N_2_O. After changing to the head-up position, HbO_2_ decreased significantly and continued to decrease for 30 minutes after the movement [45]. The recorded HbO_2_ values remained lower than the baseline even after returning to supine position [45]. A similar effect was observed in HbTot [45]. In contrast, Hb did not experience significant changes with the movement [45].

Lovell et al. [46] examined changes in blood volume with changes in position during propofol anesthesia. These investigators found increases in CBV during the head-down position that correlated with the degree of table tilt. The degree of table tilt for the head-up position was also correlated with table tilt angle, but the changes during head-up position were markedly lower than the equivalent degree of change in the head-down position [46].

**Figure 2:**
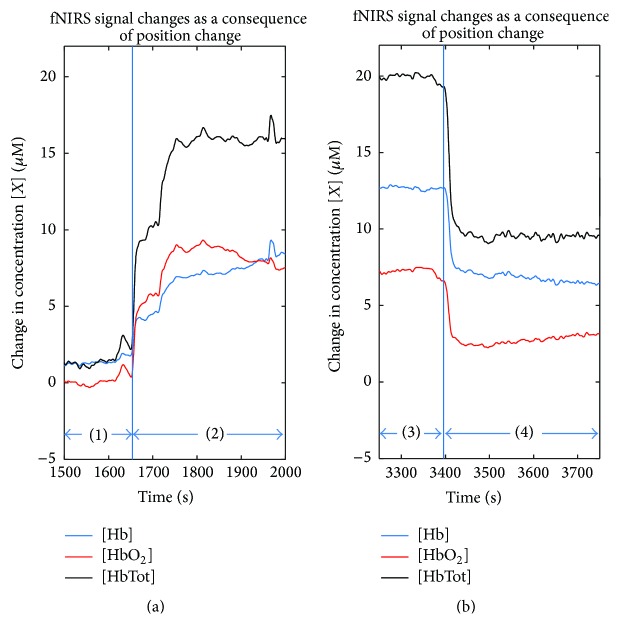
Example of the effect of changes in position on female patient (58 years old) during laparoscopic salpingo-oophorectomy using general anesthesia with 2.4% sevoflurane. (a) Response of Hb, HbO_2_, and HbTot during transition from (1) supine position to (2) Trendelenburg position. (b) Transition from (3) Trendelenburg position to (4) supine position. (G. Hernandez-Meza, personal communications, July 22, 2015, School of Biomedical Engineering, Science and Health Systems at Drexel University, Philadelphia, PA 19104).

The studies outlined in this section demonstrate that changes in position can have significant effect on fNIRS derived measures such as rSO_2_, HbTot, HbO_2_, and Hb. The changes that occur depend on the type of position and the anesthetics in use. We have observed such positional effects on hemodynamic parameters as measured by fNIRS in our prior study.  Figure 2(a) presents an example of the change that is observed on the hemodynamic parameters as a result of a transition from supine to Trendelenburg position.  Figure 2(b) shows an example of a return to supine position from Trendelenburg position.

#### 3.2.2. Effects of Other Confounders

The partial pressure of carbon dioxide (PaCO_2_) dissolved in the blood can affect the cerebral blood volume. Low levels of carbon dioxide (CO_2_) will cause a reduction in CBF. Alterations in intrathoracic pressure, such as during CO_2_ insufflation for laparoscopic procedures, can cause alterations in cerebral perfusion and PaCO_2_. A study by Owen-Reece et al. [47] showed that CBV, as measured by fNIRS, falls when the intrathoracic pressure is increased. Hyperventilation to decrease PaCO_2_ also led to a significant decrease in rSO_2_ in groups of propofol-remifentanil and sevoflurane anesthesia [48]. The evaluation by Kim et al. [49] focusing on the effect of preoxygenation with 100% oxygen found that administration of propofol with sufentanil or midazolam after preoxygenation did not have an effect on rSO_2_ values.

Another confounding element influencing the fNIRS signal is hemodilution experienced during blood loss, when the lost volume is replaced by crystalloid leading to a decrease in the volume of erythrocytes. This effect was examined via measurements of rSO_2_ during propofol and sevoflurane anesthesia [50]. During the study, oxygen saturation decreases were measured with both anesthetics [50]. Because fNIRS values are dependent on hemoglobin concentration, hemodilution during surgery can influence the hemodynamic parameters measured by fNIRS.

The effects of changes in mean arterial pressure (MAP) have also been evaluated as potential confounders of the fNIRS signal. Nissen et al. [51] evaluated rSO_2_ during blood pressure decreases that occur after IV administration of propofol and fentanyl during induction. This study found that cerebral oxygenation increased and remained stable during the surgery; however no correlation between MAP and rSO_2_ could be established [51]. The elevation in rSO_2_ during induction does however support the trends reported by other authors [34, 35].

### 3.3. Findings on fNIRS Biomarkers of Depth of Anesthesia

**Figure 3:**
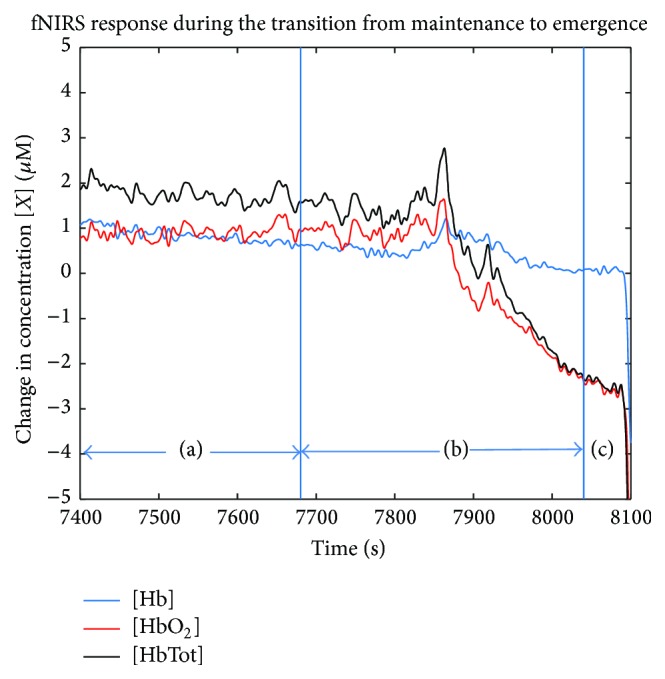
Continuous recording of fNIRS signal response during the transition from maintenance to emergence in a 62-year-old male patient. (a) Maintenance with 2% sevoflurane, (b) anesthetic removal, and (c) response 1 minute before the patient started moving. (G. Hernandez-Meza, personal communications, July 22, 2015, School of Biomedical Engineering, Science and Health Systems at Drexel University, Philadelphia, PA 19104).

Initial fNIRS studies examined the hemodynamic effects of anesthetic drugs on rSO_2_, CBV, and CBF. More recent studies have focused on finding differences in the HbO_2_, Hb, and HbTot biomarkers that could signal transitions between anesthetic states. A summary of the findings of studies included in this section can be found in Table 3. The first study to examine fNIRS biomarkers for depth of anesthesia was performed to validate the capacity of fNIRS to discriminate between “deep” and “light” anesthesia [52]. fNIRS data was collected in the operating room during abdominal or lower body surgical procedures. Deep anesthesia was classified as the 4-minute period prior to wound closure, while light anesthesia included the 4 minutes prior to eye opening. The evaluation consisted of a comparison between the levels of HbO_2_, Hb, and HbTot in 26 patients undergoing surgery with general anesthesia using either inhaled sevoflurane or desflurane [52]. The investigators established that across all prefrontal cortex PFC channels there was a statistically significant decrease in the levels of Hb during the transition from deep to light anesthesia [52]. The most significant changes were found on the right side of the PFC [52]. Similar changes were also observed in our prior study. One example of the changes in the fNIRS signal observed during the transition between maintenance and emergence is presented in Figure 3.

An additional study with a sample size of 20 patients undergoing coloproctology surgery also examined the concentration changes of Hb in the prefrontal cortex during different stages of anesthesia [53]. In the cases evaluated within this study, propofol was administered to induce anesthesia and sevoflurane was the maintenance agent. The investigators found that the average Hb concentration was significantly increased from baseline after propofol induction [53]. The comparison of average Hb concentration between deep and light anesthesia showed a decrease of Hb as the patient began to wake, and this change reached statistical significance on the right side of the PFC [53].

In a similar manner, fNIRS was used to evaluate the effects of propofol sedation during 41 outpatient elective colonoscopy procedures [54]. Due to previous results, channels located on the right PFC were selected as the PFC areas of interest and the concentrations of HbO_2_ and Hb were analyzed before and after propofol bolus infusions at different concentrations. In this study, a dose dependent increase in HbO_2_ became significant at the two- and three-minute marks after propofol administration [54]. Significant positive correlation between the change in HbO_2_ and the administered dose was also found [54]. No significant changes were found in Hb for propofol sedation doses. These results are in accordance with previous reports showing significant increases in HbO_2_ following propofol induced anesthesia [34].

## 4. Conclusions and Future Directions

Findings evidencing a reduction of the cerebral metabolic rate of oxygen as a consequence of anesthetic use in regions that included the prefrontal cortex served as motivation for the study of the sensitivity of the hemodynamic response, as measured by fNIRS, as a monitor of depth of anesthesia. Early work investigated metabolic and hemodynamic differences between anesthetics, indicating that fNIRS was capable of measuring the small changes in hemoglobin concentration that occurred in response to different anesthetics and concentrations. Although the literature on the subject is limited, fNIRS has been shown to provide information on cerebral oxygenation that is discriminative between anesthetic states [34–36, 38, 52–54], concentrations of the same anesthetic [36, 38, 52–54], and different anesthetics [34, 36].

Because the hemodynamic response measured by fNIRS is only capable of providing information on the hemoglobin species concentration it is an indirect assessment of the metabolic rate. As such, it can be affected by confounders that influence the relative quantities of Hb and HbO_2_ without changes in the metabolic rate. Position [42–46], systemic CO_2_ and O_2_ concentration [47–49], and hemodilution [50] are some of the possible confounders. Careful examination is needed to determine the hemodynamic relationships that are least affected by confounding factors or algorithms will need to be developed to mitigate their effects. Some investigators have already begun to devise algorithms to rule out possible confounders on the NIRS signal during routine oxygen saturation monitoring [55]; however, these algorithms have not yet been integrated into the monitoring software.

In the available literature only three studies were found that specifically investigated biomarkers for depth of anesthesia using the fNIRS signal. The studies by Izzetoglu et al. [52] and Leon-Dominguez et al. [53] found significant changes in Hb as the subject transitioned between deep and light anesthetic states during the use of sevoflurane and desflurane. The study by Curtin et al. [54] found that propofol prompted a dose dependent increase of HbO_2_. These findings suggest that fNIRS measured biomarkers such as Hb and HbO_2_ concentrations on the prefrontal cortex are sensitive to the effects of different anesthetic types and concentrations [52–54]. In addition, fNIRS is sensitive to different anesthetics such as propofol, sevoflurane, and desflurane at sedation and surgical doses [52–54]. Further research is needed to determine if other features of the fNIRS signal contain reliable information on the anesthetic depth. Future efforts should focus on the development of real time examination methods to assess the capacity of the fNIRS signal to provide reliable information that can be used to guide anesthesia delivery. Altogether, with its portability, affordability, safety, ease of use, and the current findings suggesting that fNIRS is indeed suitable for measuring the effects of anesthetics on the suppression of awareness and its return, this makes fNIRS attractive for further research in the field of depth of anesthesia monitoring.

## Figures and Tables

**Table 1:** Findings on the effect of anesthetics on cerebral hemodynamics.

Study	*N*	Anesthetics tested	Aim	Main findings
Lovell et al. [34]	36	Propofol, thiopental, and etomidate	To study the ability of NIRS to detect changes in cerebral oxygenation associated with the induction of anesthesia	The area under the [HbO_2_]-time curve showed significant changes with the induction of anesthetics and time. The area under the [Hb]-time curve did not significantly change with time or induction of anesthetic. The area under the [HbT]-time curve showed significant changes with respect to time and induction of anesthetic. Propofol and thiopental showed increase in the area under the curve of [HbO_2_] and [HbT] while etomidate showed a decrease of the same parameters

Fleck et al. [35]	32	Propofol	To measure cerebral oxygen and blood supply changes after propofol infusion in children with congenital heart disease	Propofol sedation caused significant increase in tissue oxygenation index while leading to significant decreases in mean arterial pressure and cardiac index

Iwasaki et al. [36]	34	Sevoflurane and propofol	To compare the induction time and changes in cerebral blood volume during induction with 8% sevoflurane, 5% sevoflurane, or propofol	HbO_2_ and HbTot were significantly greater after intubation of the 8% sevoflurane group

Valencia et al. [37]	48	Sevoflurane and propofol	To investigate differences in cerebral oxygen saturation between sevoflurane and propofol	Baseline adjusted oxygen saturation was significantly higher in the sevoflurane group

Fassoulaki et al. [38]	24	Sevoflurane and desflurane	To study the effect of different concentrations of sevoflurane and desflurane on cerebral oxygenation	At equipotent concentrations there were no significant differences in oxygen saturation between sevoflurane and desflurane. For both sevoflurane and desflurane, higher anesthetics concentrations were associated with significantly higher oxygen saturation values

Kanemaru et al. [39]	36	Midazolam, isoflurane, and aminophylline in addition to propofol	To study the influence of midazolam, isoflurane, and aminophylline on BIS and rSO_2_ when administered during propofol anesthesia maintenance	Midazolam and isoflurane administration during propofol anesthesia caused significant decreases in BIS value without affecting rSO_2_. Aminophylline caused an increase in BIS value without affecting rSO_2_

Owen-Reece et al. [40]	11 conscious subjects, 11 anesthetized subjects	Isoflurane and fentanyl	To compare measurements of CBF between healthy volunteers and anesthetized subjects (fentanyl and isoflurane). To compare measurements of CBF, during anesthesia, using fibers placed on the scalp versus the dura	A significant change in CBF was not observed as a result of anesthesia (isoflurane and fentanyl). Measurements of CBF at the dura were significantly higher than the CBF measured at the scalp

Casati et al. [41]	60	Sevoflurane	To evaluate rSO_2_ and frequency of cerebral desaturation events in healthy elderly patients during abdominal surgery	Cerebral desaturation occurred in 16 patients. Postoperative cognitive decline was observed in six patients with cerebral desaturation and six patients without desaturation. Cerebral desaturation during surgery was associated with longer hospital stays

**Table 2:** Findings on confounding factors of the fNIRS signal.

Study	*N*	Anesthetics tested	Aim	Main findings
Kim et al. [42]	27	Sevoflurane and propofol	To determine rSO_2_ differences between propofol and sevoflurane during laparoscopic surgery in the Trendelenburg position	rSO_2_ values at Trendelenburg and after Trendelenburg were significantly higher in the sevoflurane group compared to propofol. In the propofol group, rSO_2_ after Trendelenburg was significantly lower than that before Trendelenburg. Cerebral desaturation occurred in 2 propofol patients

Kim et al. [43]	40	Desflurane and propofol	To determine the effect of desflurane and propofol on rSO_2_ in the sitting position during arthroscopic shoulder surgery	rSO_2_ was higher in the desflurane group compared to the propofol group at 3, 5, 7, and 9 min after the sitting position. However, rSO_2_ decreased significantly from the baseline at the same time points after the sitting position

Closhen et al. [44]	35	Sevoflurane	To investigate changes in cerebral rSO_2_ in the beach chair position with 2 different fNIRS devices	A significant decrease in rSO_2_ after beach chair position was measured, which was reversible after return to supine position. The decrease correlated with MAP during beach chair but not during supine position

Kitajima et al. [45]	12	Sevoflurane	To determine the influence of the head-up position on Hb, HbO_2_, HbTot, Cytaa3, propofol induction, and maintenance sevoflurane + 66% N_2_O	Significant decrease in HbO_2_ was measured in the head-up position after movement and 30 minutes after. HbO_2_ remained low after return to supine position. No significant changes were measured for Hb and Cytaa3. HbTot displayed the same trend as HbO_2_

Lovell et al. [46]	20	Propofol	To measure the changes in cerebral blood volume (CBV) caused by changes in posture in awake and anesthetized subjects	CBV decreased with 18° head-up tilt and increased with 18° head-down tilt in awake subjects. In the anesthetized group there were differences between head-up and head-down tilt. In the head-down, CBV was correlated with the degree of table tilt. There was an insignificant reduction in CBV in the head-up position

Owen-Reece et al. [47]	13	Thiopentone or propofol	To evaluate the extent and duration of the hemodynamic response to an alteration in PaCO_2_ in anesthetized and healthy volunteers	CBV decreases with lower PaCO_2_. This fall in CBV is slower and smaller during anesthesia when compared to conscious subjects

Alexander et al. [48]	26	Propofol-remifentanil and sevoflurane	To examine the role of hyperventilation and systemic hemodynamic changes on the cerebral tissue oxygen saturation	Hyperventilation led to significant decreases in rSO_2_ in both the propofol-remifentanil and the sevoflurane groups. Saturation correlated significantly with etCO_2_ in both groups. Saturation also correlated significantly with MAP and CO in propofol-remifentanil group but not in the sevoflurane group

Kim et al. [49]	60	Propofol-sufentanil and midazolam	To examine the effect of induction with midazolam and propofol on oxygen supply demand balance after 100% preoxygenation	rSO_2_ increased during the preoxygenation phase compared to the baseline values. No additional increase in rSO_2_ was measured after administration of midazolam or propofol with sufentanil

Yoshitani et al. [50]	42	Propofol and isoflurane with nitrous oxide	To compare changes in rSO_2_ to changes in venous bulb oxygenation after hemodilution during propofol and isoflurane/nitrous oxide anesthesia	Mean jugular bulb O_2_ saturation was lower in the propofol than in the sevoflurane group; no significant differences were found between anesthetics in the cerebral rSO_2_. During reduction of hemoglobin concentration, jugular O_2_ saturation remained unchanged, while cerebral O_2_ saturation decreased significantly in both anesthetic groups

Nissen et al. [51]	71	Propofol-fentanyl	To examine the effect of a reduction in MAP on the rSO_2_ in the PFC during propofol-fentanyl anesthesia induction	rSO_2_ increased significantly with decreasing MAP. After induction variables recovered and remained at preanesthetic levels. No correlation between MAP and rSO_2_ could be established

**Table 3:** Findings on fNIRS biomarkers of depth of anesthesia.

Study	*N*	Anesthetics tested	Aim	Main findings
Izzetoglu et al. [52]	26	Sevoflurane and desflurane	To determine neuromarkers that can differentiate between light and deep anesthesia	Found a significant difference in Hb between deep and light anesthetic stages. The mean values indicate that light anesthesia is associated with lower Hb. The effect was seen most predominantly in the right hemisphere. Deep anesthesia was also associated with a slow rate of change in Hb, whereas light anesthesia was associated with a high rate of change

Leon-Dominguez et al. [53]	20	Propofol and sevoflurane	To examine the contribution of the human prefrontal cortex to the emergence and suppression of consciousness	Propofol causes a significant increase in Hb after induction in the left and right PFC. Removal of sevoflurane during emergence causes a significant decrease in the levels of Hb in the right PFC

Curtin et al. [54]	41	Propofol	To provide an initial evaluation into the benefits of fNIR for the monitoring of patients during GI endoscopy	fNIR can detect a dose dependent response to the infusion of propofol during a GI sedation regime. Significant increases were found in HbO_2_ concentration following propofol administration
